# *SAMD9* Is Relating With M2 Macrophage and Remarkable Malignancy Characters in Low-Grade Glioma

**DOI:** 10.3389/fimmu.2021.659659

**Published:** 2021-04-16

**Authors:** Wenping Ma, Kenan Zhang, Zhaoshi Bao, Tao Jiang, Ying Zhang

**Affiliations:** ^1^ Department of Molecular Neuropathology, Beijing Neurosurgical Institute, Capital Medical University, Beijing, China; ^2^ Department of Neurosurgery, Beijing Tiantan Hospital, Capital Medical University, Beijing, China; ^3^ Center of Brain Tumor, Beijing Institute for Brain Disorders, Beijing, China; ^4^ China National Clinical Research Center for Neurological Diseases, Beijing, China; ^5^ Chinese Glioma Genome Atlas Network (CGGA) and Asian Glioma Genome Atlas Network (AGGA), Beijing, China

**Keywords:** SAMD9, low grade glioma, immunity, macrophage, overall survival, tumor character

## Abstract

Immunoreactions regulated by TAMs (Tumor-associated macrophages) play a pivotal role in tumorigenesis and metastasis. In recent decades, treatments based on immune regulation have achieved revolutionary breakthroughs in cancer targeted therapies. The phenotypes of TAMs in gliomas are more heterogeneous and inherently complex than can be simply defined by classification into the M1 and M2 polarized states. The detailed mechanisms surrounding infiltrating macrophage phenotype and glioma characteristics remain undefined. SAMD9 (Sterile Alpha Motif Domain-Containing Protein 9) was found to be highly expressed in glioma and closely related to histological and genetic features in CGGA and TCGA databases. Simultaneously, we present evidence to show that there was a positive association between SAMD9 and malignancy characters in LGG. Univariable and Multivariate proportional hazard Cox analysis showed that SAMD9 was an independent prognostic factor for LGG. Surprisingly, Gene Ontology (GO) analysis showed SAMD9 expression level was remarkably well correlated with immunological responses and the Kyoto Encyclopedia of Genes and Genomes (KEGG) analysis supported the connection with immune responses and tumorigenesis. Immune infiltration analysis demonstrated that high SAMD9 expression resulted in an accumulation of macrophages by CIBERSORT and TIMER databases, especially positively related to macrophage total marker gene AIF1 and Macrophage M2 marker gene CD163. IHC staining further indicated a high correlation of SAMD9 with those specific macrophage markers in the immune response. Human THP-1 cells were induced into M2 macrophages, which were then co-cultured with LN229 cells. Silencing of SAMD9 by shRNA in LN229 cells attenuated the infiltration abilities of M2 macrophage. SAMD9 explored immune response via relating of M2 macrophage *in vitro*. Our results revealed SAMD9 acted as the malignancy characters in LGG, enrichment with M2 macrophage.

## Introduction

Glioma is a kind of tumor originates from glial cells or precursor cells (1–3). It is the most common type of primary tumors in the central nervous system (4–6). Traditionally, the standard treatment for gliomas consisted of total tumor surgical resection, followed by radiotherapy and concurrent chemotherapy with TMZ (temozolomide) (7–9). However, using those comprehensive complex treatments, the overall survival achieved in glioma is not more than around 15 months, indicating the need for new innovative treatments which anchor the character and heterogeneity of glioma and expose the potential vital factors affecting tumorigenesis (6). Immunoreactions regulated by the TAM play a pivotal role throughout tumor angiogenesis and metastasis, also some immunotherapies have achieved revolutionary breakthroughs in cancer targeted therapies. Therapeutics targeting the programmed cell death (PD)-1 protein and its ligands, PD-L1, such as the FDA-approved Nivolumab, an anti-PD-1 monoclonal antibody for use in melanoma, Hodgkin’s lymphoma and squamous cell lung cancer, have achieved great responses in clinical treatments (10–13). TAMs are abundant in many solid tumors characterized by diversity and plasticity, including classically M1 macrophage or M2 macrophage under different stimulation (14–16). M1 macrophages increase proinflammatory cytokine production along with the Th1 type immune response and regulate host-cell antigen presentation responses to high levels of pathogens (bacteria and viruses) and tumor cells. M2-type macrophages release anti-inflammatory factors that activate a Th2 type immune response and promote tumor growth by facilitating immune infiltration, tissue remodeling, and angiogenesis (17–20). The role of the immune response in central nervous tumors was once a controversial issue due to special structure blood-brain barrier, lacking typical lymphatic ducts and special macrophage cells (21). Although the use of immunotherapies has become a powerful strategy to effectively reduce tumor size and prolong survival in peripheral tumors (22, 23) immune response target drugs have had less dramatic benefit for glioma patients (24–28). Therefore, it was urgent and important to search for immune-related targets in gliomas.

Deleterious mutations of SAMD9 are key enabling factors for some autoimmune diseases and cancers, as well as the pathogenesis of myelodysplastic syndromes (MDS), esophageal and lung tumorigenesis (29). Our previous studies have shown that knocking down SAMD9 in glioma cells decreases glioblastoma progression (30). We reveal that elevated SAMD9 expression is closely correlated with increasing WHO grade. Furthermore, knockdown of SAMD9 attenuated the proliferation, migration and invasion of glioblastoma cells and reduced the activity of the PI3K/AKT signaling pathway (29). Till now, the detailed regulatory mechanism of SAMD9 and its impact on tumor immunity in gliomas has not been reported, whether SAMD9 could influence the immune response in gliomas is still unclear. In this paper we show that SAMD9 was elevated in low grade gliomasand acted as an indicator of poor prognosis with malignancy characteristics, IDH wild type, MGMT unmethylation and 1p/19q non-codeletion. Further analysis of glioma databases revealed that SAMD9 function was closely related to immune responses and SAMD9 enrichment was accompanied by a high M2 phenotype macrophage infiltration. The distinct correlation of SAMD9 expression levels and accumulation of macrophages implied a role for SAMD9 in governing the fate of infiltrating M2 macrophages in LGG. We verified the expression levels of SAMD9 and abundance of macrophages using clinical specimens and obtained the same trends as previously indicated by bioinformatics analysis. We further explored the molecular activity surrounding infiltrated macrophages and found SAMD9 could enhance the infiltration ability of M2 macrophage cells in vitro. In summary, our findings revealed that SAMD9 may serve as a key factor of gliomas immunity and act as an independent prognosis factor.

## Materials and Methods

### Databases and Samples

The RNA-seq data, clinical and survival information were downloaded from CGGA database (http://www.cgga.org.cn). For the TCGA validation cohort (616 patients), the RNA-seq data and corresponding clinical information were obtained from TCGA database (http://cancergenome.nih.gov/). In total, 325 samples of RNA sequencing data from CGGA mRNA sequence database 1, 616 samples of TCGA mRNA sequence database (http://cancergenome.nih.gov/), and 693 samples of RNA sequencing data from CGGA database 2 (http://www.cgga.org) were collected in this study. We retrospectively analyzed the CGGA databases which mainly included the age, grade, IDH type, MGMT promoter methylation status, TCGA molecular subtypes (31), five molecular subtypes, radiotherapy and chemotherapy characters. Simultaneously, TCGA database was included for validation, which mostly contained the same clinical features described previously. Clinical specimens were collected from glioma patients admitted for operation in Beijing Tiantan Hospital.

### Survival Prognostication

The beginning of OS was included from the initial diagnosis and the ending period was calculated to the last follow up or death. The survival data were downloaded for the CGGA and TCGA databases. All samples were divided into high and low expression groups according to the median value of SAMD9. The prognostic value of SAMD9 in these cohorts was evaluated by Kaplan-Meier with log-rank test.

### The GO and KEGG Pathway Analysis

A gene annotation and analysis resource database that detailed integrated ontology sources, including GO analysis and KEGG Pathway was carried out to evaluate the SAMD9 associated biological processes and risk score in CGGA and TCGA databases.

### Cibersort

CGGA and TCGA database were analyzed by the CIBERSORT software (https://cibersort.stanford.edu). Ten types of immune cells were evaluated in CIBERSORT to estimate the correlation of SAMD9 and infiltrating immune cells.

### TIMER Module (Tumor IMmune Estimation Resource)

Immune infiltration associated with SAMD9 expression levels in LGG were selected and visualized by TIMER Module (https://cistrome.shinyapps.io/timer/) online system.

### Cox Proportional Hazards Analysis

Univariate and multivariate Cox proportional hazards analysis were carried out to explore whether the risk score was an independent prognostic factor and screen out the most valuable independent prognostic factors.

### Immune Functions Analysis

GSVA (Gene Set Enrichment Analysis) was performed with R software as previously (31). The relationship between SAMD9 expression and immune functions was evaluated by Pearson correlation analysis. Immune function scores were calculated by GSVA analysis and the immune function gene set was downloaded from AmiGO 2 (http://amigo.geneontology.org/amigo/landing). The classification of immune functions was according to the guidelines of AmiGO 2.

### Nomogram Model Prediction

SAMD9, other partial factors such as pathological grade, age, 1p/19q status, radio status, and IDH were established using Cox regression in CGGA LGG and TCGA LGG database. Calibration curves were conducted at 1-, 3-, and 5-year time points. nomogram model was used to evaluate clinical risk score.

### Cell Culture, Lentivirus Infection, and Macrophage Polarization

Human glioma LN229 and THP-1 cells were purchased from Chinese Academy of Sciences (China), LN229 cells were cultured in DMEM medium and THP-1 cells were cultured in RPMI 1640 medium with a humidified atmosphere of 5% CO_2_ at 37°C, which containing 10% fetal bovine serum (FBS, Gibco), 100 IU/mL penicillin and 100 μg/mL streptomycin (Gibco, Thermo Scientific, NY, USA). Lentiviruses was constructed through short hairpin (sh)-RNA targeting human SAMD9 (Genepharma, Shanghai, China). LN229 cell (60%-70% density) were transfected with lentivirus vector at a multiplicity of infection of 10-15, LN229cells were infected by the viruses for 36-48 h, then they were cultured in medium containing puromycin for selecting stable SAMD9-knockdown cells. The knockdown efficiency was verified by Western blotting. the shRNA sequences were as follows (SAMD9-Homo-571, 5’-GGGTAAAGAGAACCCAGATAT-3’; SAMD9-Homo-1074, 5’-GCATTCGAGA GCCAAGATTTG-3’; non-targeting control, 5’-TTCTCCGAACG TGTCACGT-3’). To polarize M0 macrophages, THP-1 cells (2.5 × 10^5^) were plated into 6-well plate and treated with 320 nM phorbol 12-myristate 13-acetate (PMA; Sigma, St. Louis, MO, USA) at 37°C for 6h. To polarize M2 macrophages, then added M2-polarizing reagents (20 ng/ml IL-4 plus 20 ng/ml IL-13), incubated at 37°C for 72 h (32–35).

### M2 Macrophage Infiltration Assays

M2 macrophage infiltration assays were conducted by seeding 2.5×10^5^ M2 macrophage cells (300μl) without serum for 12 h in the upper chamber of a Transwell plate (size 5μm, Corning, NY, USA). 2.5×10^5^ LN229 glioma cells were cultured with10% FBS in bottom plate(700μl), each experiment. After incubation at 37°C for 24 h, the cells in the upper chamber were fixed in 4% formalin and stained with 0.1% crystal violet. The infiltrated M2 macrophage cells were counted in three randomly selected fields from each membrane and each experiment was performed three times (29, 36–38).

### Semi-Quantitative Immunohistochemistry

Paraffin sections were made as previously described (39, 40). After deparaffinization, sections were immersed in 100% ethanol, 96% ethanol, and 75% ethanol, and subjected to heat-induced antigen retrieval at 120°C for 10 min, After cooling to room temperature, each slide was incubated with the primary antibody overnight at 4C, then incubated with the secondary antibody for 1 h. Color development was produced using DAB staining for 5 min and hematoxylin counterstained for 1 min. In our research, we stained the serial sections of the same glioma tissue and observed in the same visual field. Positive expression of SAMD9 was located in cytoplasm of tumor cells. SAMD9 and other protein expression levels were evaluated independently by two experienced pathologists using the following method. A: Cell staining intensity (at 10x20 magnification, 3 different fields of view were observed and the average counts of 3 fields taken), the count scoring was as follows: negative staining, 0 point; weakly positive staining, 1 point; positive staining but with light brown background, 2 points; positive staining without background, 3 points. B: Area staining intensity (at 10 x 4 magnification the total positive area was observed and evaluated): positive area = 0%, 0 point; positive area =1%-25%, 1 point; positive area = 26%-50%, 2 points; positive area = 51%-75%, 3 points; positive area>75%, 4 points. C: The degree of positive staining for each section was determined by multiplication of the values for A and B: 1-3 was classified as weakly positive (+); 4-6 as positive (++); and 7-12 as strongly positive (+++).

### Other Analysis

Heatmap was plotted by R-CRAN-Package pheatmap through Raivo Kolde·GitHub (http://cran.r-project.org), Before data entry, the data of CGGA and TCGA was applied with log transformation by mean value of gene and normalized the rows of those data.

### Statistical Analysis

Student’s t-test or the chi-squared test were performed to clarify the differences in clinicopathological characteristics among these samples. p < 0.05 was considered statistically significant. correlation analysis of various factors and graphic work were accomplished by R language (Version 4.0.2) and SPSS (SPSS Inc., Chicago, Ill., USA).

### Ethics

We confirmed that the research involving experiments on human subjects met the ethical standards of the Helsinki Declaration in 1975. The research was approved by the ethics committee of Beijing Tiantan Hospital, Capital Medical University, and all patient and their relatives had provided written informed consent.

## Results

### SAMD9 Expression is Significantly Correlated With Malignancy Degree and Subtype Feature in Lower Grade Gliomas

**Figure 1:**
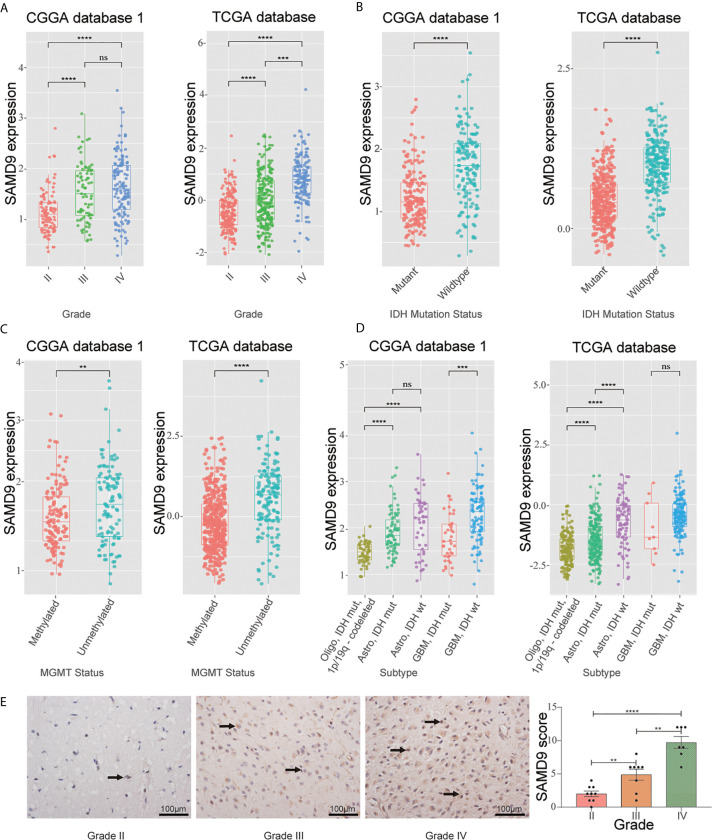
WHO grade, IDH1 status, MGMT promoter methylation status and transcriptional characteristic subtype relative to SAMD9 expression. **(A)** The correlation of SAMD9 expression level with WHO grade. SAMD9 expression levels in glioma of WHO grade II-IV in CGGA database 1 and TCGA database, the difference between III & IV in TCGA database. **(B)** The relationship between *SAMD9* transcription level and *IDH1* mutation in CGGA and TCGA databases. **(C)** The correlation of *SAMD9* expression level and *MGMT* promoter methylation status, the difference in CGGA database 1 and in TCGA database. **(D)** The relationship between *SAMD9* transcription level and *IDH1* mutation in each transcriptional characteristic subtype in CGGA and TCGA databases. **(E)** SAMD9 expression in different grades of gliomas (Grade II, n=7; Grade III, n=8; Grade IV, n=9) detected by IHC analysis. Scale bar = 100 mm. **, ***, **** and ns indicate *p* < 0.01, *p* < 0.001, *p* < 0.0001 and no significance, respectively.

To further explore the role of SAMD9 in the malignant progression of glioma, we analyzed its expression levels in different grades in CGGA dataset 1, TCGA dataset, and CGGA dataset 2 of glioma. SAMD9 expression levels increased along with grade II to III progression very significantly in glioma (**Figure 1A**), it also showed an increasing grade III to IV tendency in the TCGA database (**Figure 1A**). We also detected the expression of SAMD9 in clinical glioma specimens and observed that SAMD9 was indeed increased with tumor grade and increased in high-grade gliomas (**Figure 1E**). IDH mutation is a principal driver gene in low grade gliomas, with an incidence of more than 70% (41). we therefore explored the relationship between SAMD9 expression and the status of *IDH*. In both the CGGA database 1 and TCGA database, patients with high SAMD9 expression were synchronized with wild type *IDH*, whereas most of those with low SAMD9 expression associated with the *IDH* mutation status (**Figure 1B**). The alkylating drug TMZ is routinely used for chemotherapy in glioma patients and *MGMT* promoter status was identified as a useful predictive biomarker for TMZ efficacy (42). We assessed the SAMD9 transcription level and the status of *MGMT* promoter methylation in both the CGGA sequence database 1 and TCGA sequence database. Patients with lower SAMD9 expression were found to have the *MGMT* promoter methylated, conversely, those with high SAMD9 expression associated with the unmethylated *MGMT* promoter (**Figure 1C**). The correlation between SAMD9 expression and glioma subtypes could also reflect SAMD9 function attributed to human glioma characteristics. We also systematically characterized the molecular features of classified lower grade gliomas through IDH and 1p/19q status. Patients with high expressions of SAMD9 were more concentrated in the astrocytoma than oligodendrocyte type. This information is highly consistent across the CGGA database 1 and TCGA databases (**Figure 1D**). These results indicated that lower expression of SAMD9 may be correlated with better prognosis of gliomas. we also achieved the same tendency characters of SAMD9 in CGGA database 2 (**Supplementary Figure 1**).

### Landscape of the Correlations of SAMD9 Accompanied With Classical Genetic Alterations and Clinical Character of Glioma

**Figure 2:**
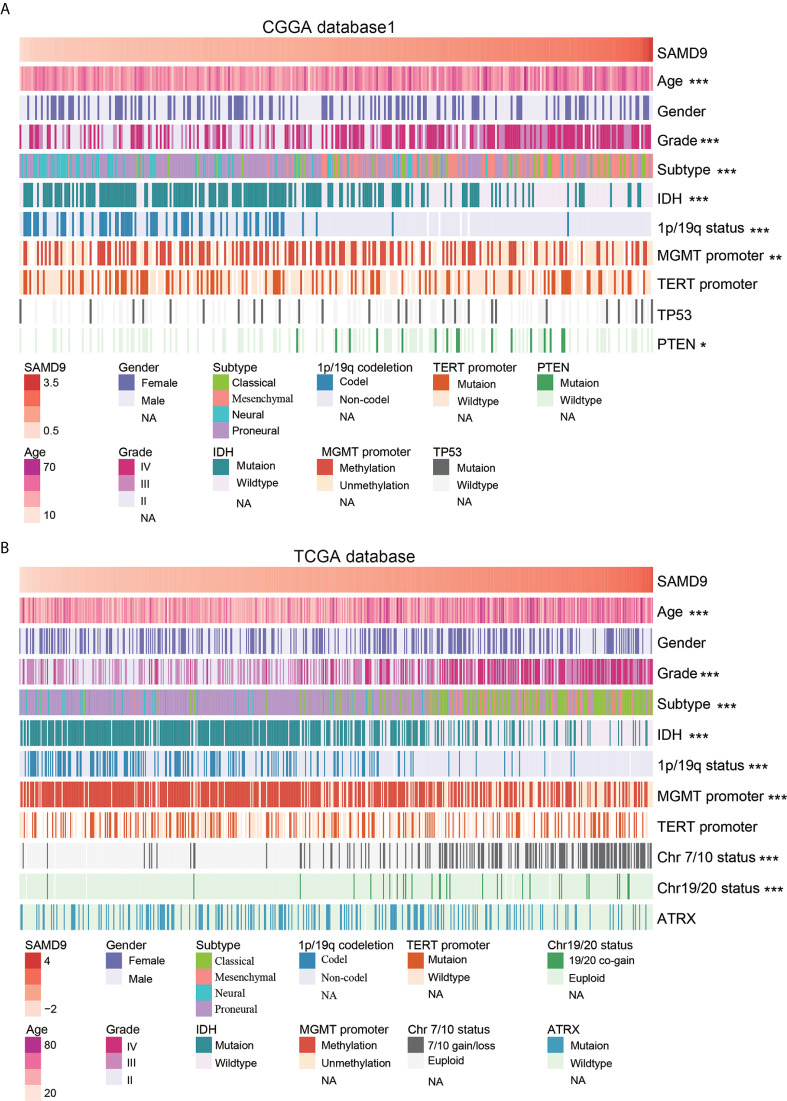
SAMD9 transcription level accompanied with classical genetic alterations and clinical character of glioma. **(A)** With increasing expression of SAMD9 in CGGA database 1, patients characterized with age group, ***high grade, ***oligodendrocyte and GBM subtype, ***IDH wild type, ***1p/19q intact, ***MGMT promoter unmethylation status, **PTEN mutation. **(B)** The correlation of *SAMD9* expression level in TCGA database with age group, ***high grade, ***oligodendrocyte and GBM subtype, ***IDH wild type, ***1p/19q intact ***MGMT promoter unmethylation status, ***Chromosome 7/10 Euploid, ***Chromosome 19/20 Euploid, *, **, and *** indicate *p* < 0.05, *p* < 0.01, *p* < 0.001, respectively.

Oncoprint plots were used to visualize the correlation between SAMD9 expression level and classical genetic alterations in CGGA and TCGA dataset by complex Heatmap package. In CGGA dataset 1, we noticed that with increasing expression of SAMD9, patients tended towards a higher grade, *IDH* wild type, intact 1p/19q, *PTEN* mutation, and *MGMT* promoter unmethylation. We obtained more reliable and robust results with the large sample TCGA database. Furthermore, *MGMT* promoter unmethylation and fewer *ATRX* mutations appeared frequently in SAMD9 high expressing patients. Even more remarkable was a gain of chromosome 7 and loss of chromosome 10 in those patients. The high expression of SAMD9 showed a consistent trend with the typical malignant genetic features disclosing malignancy characters in gliomas. The high expression of SAMD9 synchronized with malignancy characters indicate the oncogenic nature of SAMD9 which may play an important role in the biological process of tumorigenesis (**Figure 2** and **Tables 1**, **2**).

**Table 1:** Clinicopathological of SAMD9 expression in CGGA database 1.

Variable	Group	Total	SAMD9 Expression		*p*-value
		325	Low(-+)	High(++)	
			180	145	
Age					
	<=43	183	119	64	1.15E-04
	>43	142	61	81	
Gender					
	0	203	113	90	9.88E-01
	1	122	67	55	
Grade					
	II	103	86	17	6.25E-11
	III	79	37	42	
	IV	139	57	82	
Subtype					
	Classical	32	10	22	7.63E-15
	Mesenchymal	78	17	61	
	Neural	57	47	10	
	Proneural	158	106	52	
IDH1					
	0	149	48	101	3.42E-14
	1	175	131	44	
1p/19q status					
	0	250	112	138	6.15E-14
	1	67	65	2	
MGMT promoter					
	0	110	48	62	6.45E-03
	1	134	83	51	
TERT promoter					
	0	169	93	76	5.42E-01
	1	92	55	37	
TP53					
	0	89	40	49	9.42E-01
	1	27	13	14	
PTEN					
	0	100	52	48	2.76E-02
	1	16	3	13	

**Table 2:** Clinicopathological of SAMD9 expression in TCGA database.

Variable	Group	Total	SAMD9 Expression		*p*-value
		601	Low(-+)	High(++)	
			317	284	
Age					
	<=43	266	185	81	3.59E-13
	>43	335	132	203	
Gender					
	0	351	193	158	2.22E-01
	1	250	124	126	
Grade					
	II	211	162	49	1.13E-28
	III	236	129	107	
	IV	154	26	128	
Subtype					
	Classical	149	20	129	3.23E-40
	Mesenchymal	35	1	34	
	Neural	36	31	5	
	Proneural	381	265	116	
IDH1					
	0	227	46	181	5.40E-35
	1	374	271	103	
1p/19q status					
	0	446	191	255	7.82E-18
	1	149	125	24	
MGMT promoter					
	0	147	45	102	3.88E-11
	1	420	263	157	
TERT promoter					
	0	162	96	66	4.26E-01
	1	155	84	71	
ATRX					
	0	422	215	207	1.37E-01
	1	174	101	73	
Chr19/20 status					
	0	564	311	253	6.10E-06
	1	29	3	26	
Chr7/10 status					
	0	448	294	154	4.61E-27
	1	145	20	125	

### SAMD9 mRNA Expression Levels Predicted Overall Survival in All Grade Glioma and Especially in Lower Grade Glioma Patients

**Figure 3:**
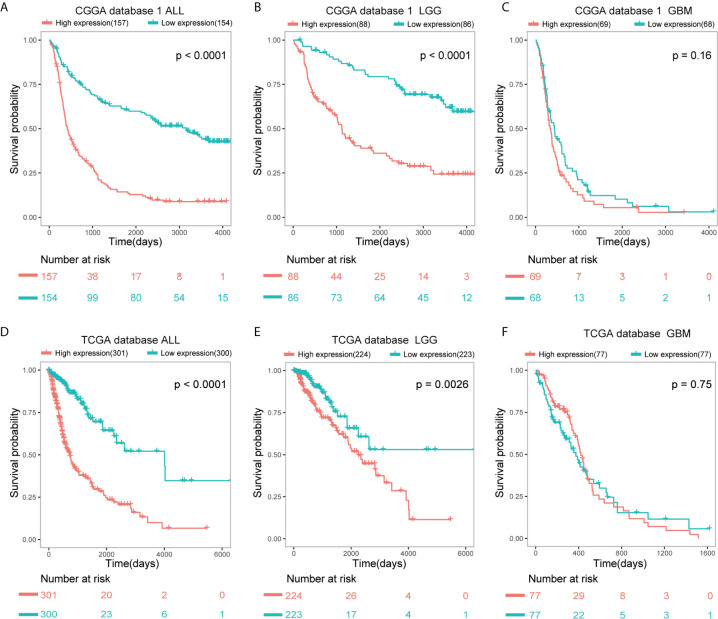
Patients with higher SAMD9 level exhibit poorer OS. **(A)** Half of the patients with higher SAMD9 expression exhibited shorter OS in Kaplan-Meier analyses based on CGGA database 1 ALL, *p* < 0.0001. **(B)** The half of patients with higher SAMD9 expression exhibited shorter OS in Kaplan-Meier analyses based on CGGA database 1 LGG, *p* < 0.0001. **(C)** Half of patients with higher *SAMD9* expression exhibited shorter OS in Kaplan-Meier analyses based on CGGA database 1 GBM, *p* = 0.16. **(D)** Half of the patients with higher SAMD9 expression exhibited shorter OS in Kaplan-Meier analyses based on TCGA database ALL, *p* < 0.0001. **(E)** The half of patients with higher SAMD9 expression exhibited shorter OS in Kaplan-Meier analyses based on TCGA database LGG, *p* = 0.0026. **(F)** Half of patients with higher *SAMD9* expression exhibited shorter OS in Kaplan-Meier analyses based on TCGA database GBM, *p* = 0.75.

SAMD9 expression level was sufficient to predict OS (overall survival) of patients with glioma in three datasets. The patients of all glioma, LGG and glioblastoma (GBM) were divided into two groups on the basis of Grade type derived from CGGA database 1 (**Figures 3A–C**). Patients with higher SAMD9 expression exhibited shorter OS in Kaplan-Meier analyses in all glioma and LGG (**Figure 3A** *p* < 0.0001; **Figure 3B** *p* < 0.0001). To avoid the possible bias caused by a single database, we further expanded sample volumes in the TCGA database and confirmed the true character of SAMD9, we revealed the OS analyses with SAMD9 expression level derived from TCGA dataset (**Figures 3D–F**). The half of patients with higher SAMD9 expression exhibited shorter OS in either all grades (**Figure 3D** *p* < 0.0001) or LGG (**Figure 3E** *p* = 0.0026), but not in GBM (**Figure 3F** *p* = 0.75). We also got the same trend in CGGA mRNA sequence database 2 and the OS distinguishing effects of SAMD9 expression level were very significant in all glioma and LGG (**Supplementary Figure 2A** *p* < 0.0001; **Supplementary Figure 2B** *p* = 0.0002).

### Univariate and Multivariate Analysis Showed SAMD9 Together With Other Related Clinicopathological Factors of Prognostic Significance

**Figure 4:**
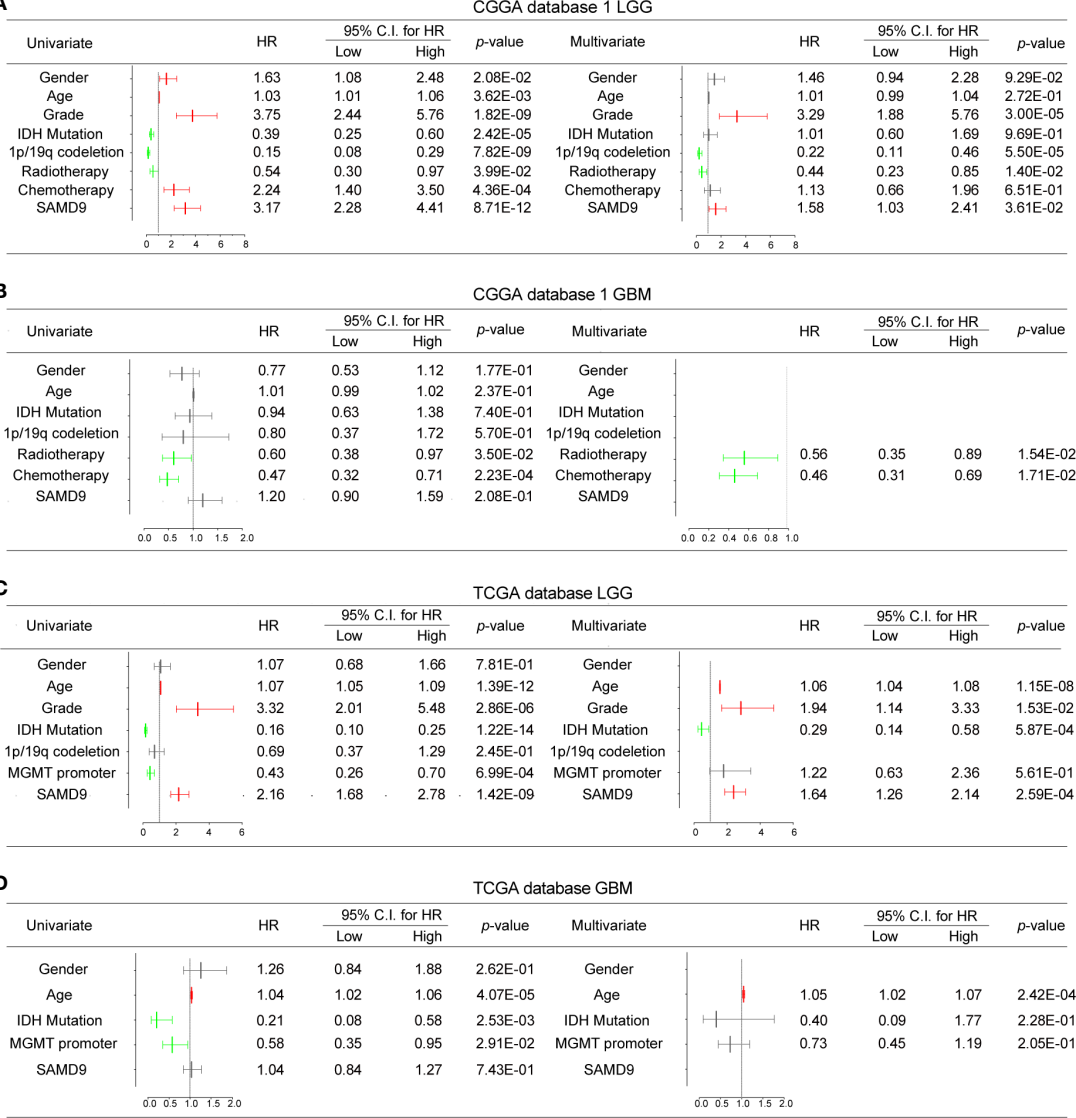
Univariate and Multivariate Cox regression analyses and correlations with classic genetic alterations of SAMD9. **(A)** Univariate and Multivariate regression analyses of SAMD9 expression level and several related clinical variables in CGGA database 1 LGG, Red color indicates protective factor HR > 1, Green color indicates harmful factors HR < 1, Gray color represents HR crossing 1. HR of SAMD9 and Grade > 1, *p* < 0.0001. HR of 1p/19q codeletion < 1, *p* < 0.0001. HR of Radiotherapy < 1, *p* < 0.0001. **(B)** Univariate and Multivariate regression analyses of SAMD9 expression level and several related clinical variables in CGGA database 1 GBM, HR of Radiotherapy < 1, *p* = 0.0154. HR of 1p/19q codeletion < 1, *p* =0.0171. **(C)** Univariate and Multivariate regression analyses of SAMD9 expression level and several related clinical variables in TCGA database LGG. HR of SAMD9 > 1, *p* < 0.0001. HR of Grade > 1, *p* = 0.0153. HR of Age > 1, *p* < 0.0001. **(D)** Univariate and Multivariate regression analyses of SAMD9 expression level and several related clinical variables in TCGA database GBM. HR of Age > 1, *p* < 0.0001.

To further explore SAMD9 function in different grades and determine whether the risk score was an independent and significant prognostic factor in glioma, we carried out univariate and multivariate Cox regression analyses in the above databases. In addition, univariate and multivariate Cox regression analyses showed SAMD9, together with classical malignancy features of glioma (gender, age, WHO grade, *IDH* status, 1p/19q status, *MGMT* promoter status, radiotherapy, and chemotherapy). The univariate and multivariate Cox regression analysis in CGGA database1 LGG identified SAMD9 expression (univariate hazard ratio (HR): 3.17, *p* = 8.71E-12; multivariate HR: 1.58, *p* = 3.61E-02), WHO grade (univariate HR: 3.75, *p* = 1.82E-09; multivariate HR: 3.29, *p* = 1.82E-09), 1p/19q codeletion (univariate HR: 0.15, *p* = 7.82E-09; multivariate HR: 0.22, *p* = 5.50E-05), radiotherapy (univariate HR: 0.54, *p* = 3.99E-02; multivariate HR: 0.44, *p* = 1.40E-02) (**Figure 4A**). We discovered SAMD9 together with WHO grade (red color) in LGG may serve as independent risk factors but irrelevant in GBM (**Figure 4B**), 1p/19q codeletion and radiotherapy were designated as protectable variances. We also analyzed *SAMD9* and the related clinical features in TCGA LGG database and got consistent trends. SAMD9 expression (univariate HR: 2.16, *p* = 1.42E-09; multivariate HR: 1.64, *p*=2.59E-04), WHO Grade (univariate HR: 3.32, *p* = 2.86E-06; multivariate HR: 1.94, *p* = 1.53E-02) and age (univariate HR: 1.07, *p* = 1.39E-12; multivariate HR:1.06, *p* = 1.15E-08), *IDH* mutation (univariate HR: 0.16, *p* = 1.22E-14; multivariate HR:0.29, *p* = 5.87E-04) (**Figure 4C**). We found *SAMD9* together with grade and age (red color) had clinical significance in LGG but evaluated no significance in GBM (**Figure 4D**), IDH mutation was a protective factor. Further similar trend results were obtained in CGGA database2 (**Supplementary Figures 3A, B**). In summary, SAMD9 may act as an independent prognostic factor in low-grade gliomas.

### Nomogram Model Predicted the Overall Survival and Integrated Clinic Pathologic Risk Score

**Figure 5:**
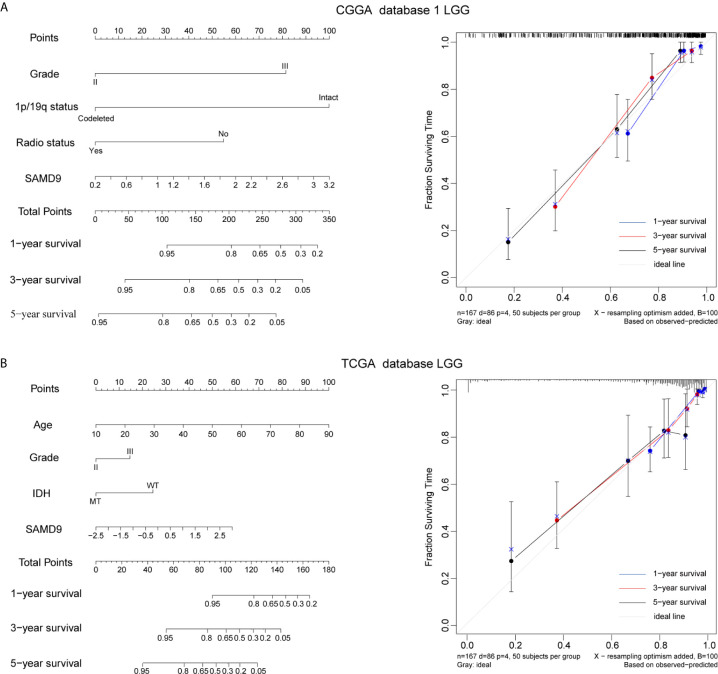
Nomogram prediction model for overall survival (OS) and other integrated risk factors. **(A)** A nomogram was developed by integrating the SAMD9 expression with the clinicopathologic features, grade, 1p/19q codeletion status, radiotherapy status in the CGGA database 1 (left). Calibration plot of nomogram for predicting OS at 1- (blue line), 3- (red line) and 5- (black line) years, ideal line as control (white line) in the CGGA database 1 (right). **(B)** A nomogram was developed by integrating the SAMD9 expression with the clinicopathologic features, age, grade, IDH status (MT, mutant. WT, wild type.) in the TCGA database (left). Calibration plot of nomogram for predicting OS at 1- (blue line), 3- (red line) and 5- (black line) years, ideal line as control (white line) in the TCGA database LGG (right).

The clinical prognostic factors for overall survival were identified and incorporated to construct nomograms for 1-, 3- and 5-year overall survival, respectively (**Figures 5A, B** **)**. These nomograms can easily be used by providers to estimate a patient’s prognosis; the only clinical details a provider needs to use these nomograms effectively are grade, 1p/19q codeletion status, radiotherapy status and SAMD9 expression levels. The calibration plot for the probability of survival at 1-, 3- and 5-years also showed optimal concordance with the prediction in the TCGA validation cohort. The C-indices respectively were 0.81 in CGGA database 1 LGG (**Figure 5A**) and 0.87 in TCGA database LGG (**Figure 5B**). We got an accurate and reliable 1-, 3- and 5-year predicting survival of glioma patients through these nomogram-based results. In addition, a calibration plot for probability of survival also showed satisfactory concordance with the prediction of 1-, 3-, and 5-year OS in CGGA database 2 (**Supplementary Figure 4**).

### Functional Enrichment Analysis of Tightly Correlated Genes With SAMD9 in LGG

**Figure 6:**
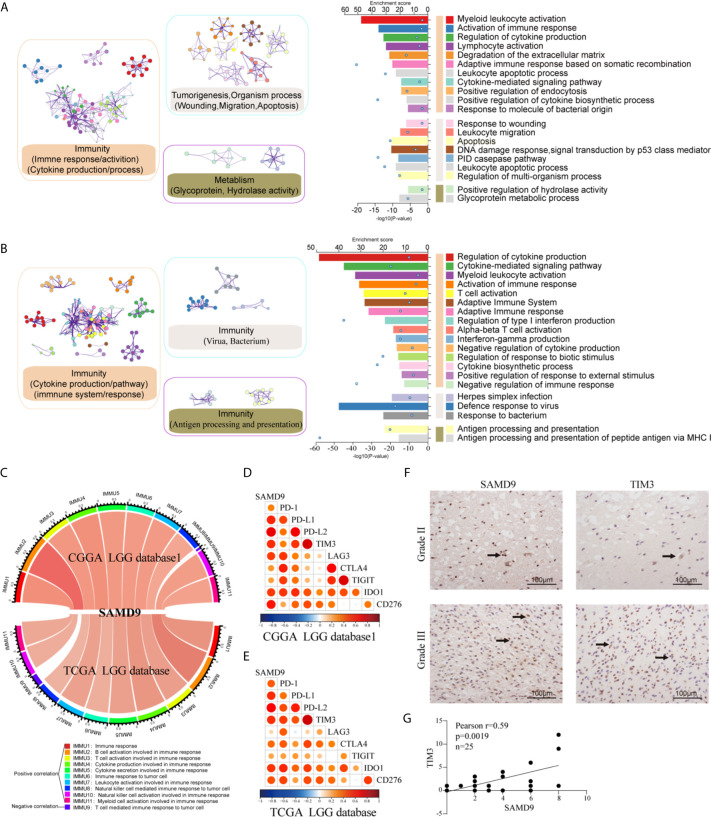
Functional analysis of SAMD9 correlated genes relative to immune response. **(A, B)**. Functional enrichments of SAMD9 related genes (R > 0.5) in CGGA database 1and CGGA database, Cluster showed the possible functions of TOP 20 genes terms and description annotated the detailed pathways. Blue dots represent the enrichment scores of difference terms in the upper axis, the *P*-value of difference terms in the lower axis, all analysis was calculated by Metascape online. **(C)** The correlation coefficient between SAMD9 and different immune cells in CGGA and TCGA databases. IMMU9 represent a negative correlation and other IMMNUs represent a positive correlation. **(D, E)** Correlation Analysis of SAMD9 and nine immune-related checkpoints (PD-1, PD-L1, PD-L2, TIM3, LAG3, CTLA4, TIGIT, IDO1, CD276) in CGGA database 1 LGG and TCGA database LGG. Color depth and circle square represent the degrees of correlation. **(F)** SAMD9 and TIM3 expression levels in glioma specimens determined by IHC analysis in grade II and III. Scale bar = 100 μm. **(G)** Correlation analysis of SAMD9 and TIM3 immunohistochemical scores. X-axis, SAMD9 staining score; y-axis, TIM3 staining score; correlation coefficient r = 0.59; p = 0.0019.

We analyzed related genes that tightly correlated with the SAMD9 expression levels of each sample in CGGA and TCGA databases (R > 0.5) by Pearson correlation analysis, then further explored the biological function of those related genes, and used the DAVID online system to annotate those significant enrichment functions. The enrichment results indicate different terms in the positive-expression. Comparisons of the TOP20 gene terms, mostly focused on the immune response and tumorigenesis in CGGA database1 LGG (twelve related functions) (**Figure 6A** and **Supplementary Table 1**) and were almost totally immune response related in the TCGA database LGG (fourteen functions) (**Figure 6B** and **Supplementary Table 2**). Through these GO and KEGG pathways validations, we strongly speculate that SAMD9 was directly correlated with immunological responses. Positive function analysis revealed activation of the immune response, myeloid leukocyte activation, regulation of cytokine production, lymphocyte activation, defense response to virus, cytokine-mediated signaling pathway, myeloid leukocyte activation, activation of immune response, T cell activation, Adaptive Immune System and regulation of type I interferon production. The top 5 molecular functions were located in immune response, cytokine production, defense response to virus. This founding illustrates that SAMD9 has a strong correlation with the immune system. To understand the role of SAMD9 in the immune system, we performed a correlation coefficient analysis on data from the CGGA database1 LGG and the TCGA database LGG databases (**Figure 6C** and **Tables 3**, **4**). We observed that almost all of immune functions showed positive correlation with SAMD9; only the term of “T cell-mediated immune response” was found to be negatively correlated with SAMD9.To further explore the relationship between SAMD9 and immune responses, we detected nine immune-related checkpoints by Pearson correlation analysis and found that SAMD9 is positively associated with TIM3, CD276, and IDO1, these evaluations indicating SAMD9 maybe a potential antitumoral target by inhibiting these checkpoint proteins(**Figures 6D, E**). We also detected the SAMD9 and TIM3 protein levels in 20 LGG patients by IHC and found that SAMD9 expression positive correlated with TIM3 expression (**Figures 6F, G** r = 0.59, p = 0.0019).

**Table 3:** SAMD9 and related immune scores in CGGA database 1 LGG.

SAMD9	IMMU1	IMMU2	IMMU3	IMMU4	IMMU5	IMMU6	IMMU7	IMMU8	IMMU9	IMMU10	IMMU11
r	0.553098	0.677422	0.514613	0.543303	0.50371	0.493287	0.497261	0.439126	0.036262	0.483541	0.46568

**Table 4:** SAMD9 and related immune scores in TCGA database LGG.

SAMD9	IMMU1	IMMU2	IMMU3	IMMU4	IMMU5	IMMU6	IMMU7	IMMU8	IMMU9	IMMU10	IMMU11
r	0.507254	0.594908	0.456405	0.496002	0.446906	0.383906	0.444729	0.364097	-0.18261	0.385898	0.410394

### SAMD9 Relating M2 Macrophages in LGG and Enhances the Infiltration of M2 Macrophages *In Vitro*

**Figure 7:**
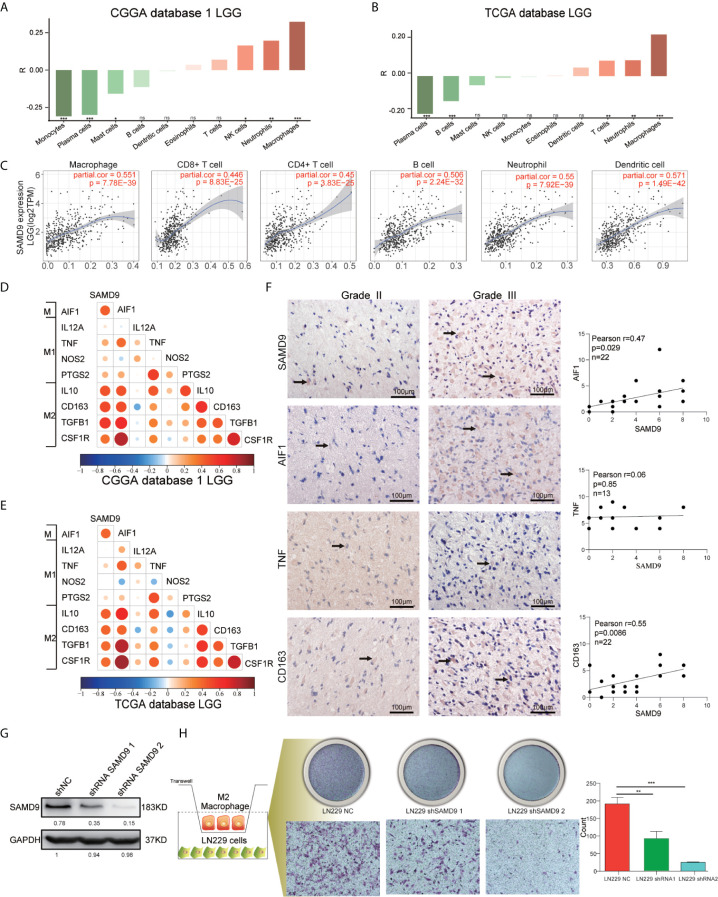
SAMD9 enrichment with M2 macrophages in LGG. **(A, B)** The correlation between SAMD9 and ten types of infiltrating immune cells (B cells, plasma cells, T cells, NK cells, monocytes, macrophages, dendritic cells, mast cells, eosinophils, neutrophils) in CGGA database 1 LGG, Correlation between SAMD9 and macrophages in CGGA database 1 LGG (R = 0.32, *p* = 9.6E-06) and in TCGA database LGG (R = 0.34, *p* = 1.1E-13). **(C)** Correlation between SAMD9 and the six immune cell types (B cells, CD4 T cells, CD8 T cells, neutrophils, macrophages and dendritic cells) on TIMER algorithm, macrophages, partial.cor = 0.551, *p* = 7.78E-39. **(D, E)** Nine classical phenotype markers of M (*AIF1*), M1 (*IL12A*, *TNF, NOS2, PTGS2*) and M2 (*IL10*, *CCL163*, *TGFB1, CSF1R)* were analyzed in CGGA and TCGA database, Color depth and circle square represent the degrees of correlation. **(F)** SAMD9, AIF1, CD163 and TNF expression levels in glioma specimens determined by IHC analysis in grade II and III. Scale bar = 100 μm. Correlation analysis of SAMD9 and related proteins immunohistochemical scores. X-axis, SAMD9 staining score; y-axis, AIF1, CD163 and TNF staining score; Correlation coefficient between SAMD9 and AIF1, r = 0.47; *p* = 0.029. Correlation coefficient between SAMD9 and TNF, r = 0.06; *p* = 0.85. Correlation coefficient between SAMD9 and CD163, r = 0.55; *p* =0.0086. **(G)** Western blots of LN229 cells with stable expression of shSAMD9. **(H)** Infiltration of M2 macrophage in LN229 NC (left), LN229 shSAMD9 1 (middle), LN229 shSAMD9 2 (right). LN229 NC & LN229 shSAMD9 1, **. LN229 NC & LN229 shSAMD9 2, ***.*, **, ***, **** and ns indicate *p* < 0.05, *p* < 0.01, *p* < 0.001, *p* < 0.0001 and no significance, respectively.

All the above results confirmed that SAMD9 may serve as an independent influencing factor for malignancy of LGG, poor prognostic survival and enriched with immune related biological progress (myeloid leukocyte activation, activation of immune response, regulation of cytokine production, defense response to virus, immune checkpoints) that regulate glioma character phenotype.To further explore the cells that have the remarkable significant impact on SAMD9, we evaluated the classical proportions of 10 types of infiltrating immune cells (B cells, plasma cells, T cells, NK cells, monocytes, macrophages, dendritic cells, mast cells, eosinophils, neutrophils) using the Cell-type Identification by Spearman’s rank correlation test in both CGGA and TCGA datasets. Among these immune cells, we found that macrophages significantly infiltrated in LGG gliomas and were highly consistent with SAMD9 expression in both CGGA database 1 LGG and TCGA databases LGG. The abundance of macrophage was correlated with LGG glioma in those databases (**Figures 7A, B**) (R = 0.32, *p* = 9.6E-06; R = 0.34, *p* = 1.1E-13). The partial correlation between the SAMD9 expression level and the six immune cell types: B cell, CD4 T cell, CD8 T cell, neutrophils, macrophages and dendritic cells in the tumor microenvironment was systematically estimated based on the Tumor Immune Estimation Resource (TIMER) algorithm. there was a highly significant correlation between measurements of macrophage infiltration and SAMD9 expression in LGG (**Figure 7C**) (partial.cor = 0.551, *p* = 7.78E-39). Although five other immune cell types showed significantly in LGG, they did not match the data in CIBERSORT. In conclusion, samples with higher *SAMD9* expression exhibited apparent concordance with encirclement of macrophage cells. The classical phenotype markers of M (*AIF1*), M1 (*IL12A*, *TNF, NOS2, PTGS2*) and M2 (*IL10*, *CCL163*, *TGFB1, CSF1R)* were analyzed in CGGA and TCGA database and found *SAMD9* had stronger positive correlation with M (*AIF1*) and M2 (*IL10*, *CCL163*, *TGFB1, CSF1R)* markers, but inconsistent correlation with M1 (*IL12A*, *TNF, NOS2, PTGS2*) markers (**Figures 7D, E**). Furthermore, we examined the expression levels of related proteins in clinical samples using IHC staining and performed correlation analysis of SAMD9 and those markers. Detailed antibody information in **Table 5**. The results showed that there was higher staining intensity of macrophage total marker AIF1 and M2 marker CD163in WHO grade III than grade II and significantly correlated with SAMD9 (Total Macrophage and SAMD9 Pearson r = 0.47, *p* = 0.029. Macrophage M2 and SAMD9 Pearson r = 0.55, *p* = 0.0086). but M1 marker TNF expressed lower grade III than grade II and showed no significant correlation with SAMD9 (Total Macrophage & SAMD9 Pearson r = 0.06, *p* = 0.85) (**Figure 7F**). To explore the effects of SAMD9 -on M2 macrophage migrative activity, we stably knocked down SAMD9 expression level in LN229 gliomas cells (**Figure 7G**). THP-1 cells were differentiated into M2 macrophage according to classical inducing methods (32, 33). Stable silencing of SAMD9 in LN229 cells reduced the infiltration of M2 macrophage (NC group & shSAMD9 1, *p* <0.01; NC group & shSAMD9 2, *p* <0.001) (**Figure 7H**).

**Table 5:** Detailed antibody information.

Antibody	Number	Antibody dilution
Anti-SAMD9	(ab121664)	1:200
Anti-TNF	(ab270264)	1:150
Anti-TIM3	(ab241332)	1:200
Anti-CD163	(ab189915)	1:200
Anti-AIF1	(ab204493)	1:200

## Discussion

New strategies for immune targeted cancer therapy could be anchored in specifically interfering with the M2-like TAM signaling cascade pathway or switching polarization of tumor-promoting M2-like TAMs to a tumoricidal M1-like phenotype. The greater infiltration of TAMs is proportionally correlated with a long term of negative prognosis, as observed in experimental animals and clinical research (43–45). TAMs play major roles in tumor progression and it was widely recognized that the M2 phenotype provided an advantage to high grade gliomas (46, 47). There was a fierce dispute about the macrophage polarization state and the proportions of M1 versus M2 in LGG because of the prolonged dynamic stable state of low-grade gliomas (47–49).

SAMD9 expression levels maybe a robust index for the evaluation of the degree of the immune response, deleterious mutations of SAMD9 is the cause of some autoimmune diseases and cancers (50, 51). The IFN-γ binding element is located within the SAMD9 promoter in humans and IFN is the cytokine produced by gliomas, influencing the immune response through TAMs (52, 53). We have revealed that knocking down SAMD9 expression levels in glioma cells decreased the glioblastoma cell progression via the AKT/PI3K pathway, but the detailed mechanism of how SAMD9 affected the occurrence of gliomas and its impact on tumor immunity has not been reported in gliomas (29).

This is the first report to demonstrate that SAMD9 has a role not only as a significance marker related to malignancy characteristics in glioma, but also may as an independent prognostic indicator in lower grade glioma patients; further study found that SAMD9 influences the immune response by increasing the ability of M2 macrophage to infiltrate *in vitro*.

LGG is a transient dynamic quiescence state which almost always invariably develops into secondary glioblastoma (sGBM) (54, 55). Many tumor-related events occur prior to reaching this stage, which potentially provides an optimal intervention window for glioma. Therefore, an urgent strategy is now needed to exploit the novel factors involved in macrophage function in tumorigenesis and metastasis. Revealing the molecular function of SAMD9 will determine its clinical application. SAMD9 may be a diagnostic or prognostic indicator for low grade glioma and also a new potential therapeutic target for treating gliomas. A better understanding of the role of SAMD9 in LGG and its detailed mechanism could certainly open up a new avenue for anti-glioma therapy. Future research will focus on the exploration of SAMD9 specific inhibitors and evaluating their therapeutic effect in gliomas.

## Data Availability Statement

The datasets presented in this study can be found in online repositories. The names of the repository/repositories and accession number(s) can be found in the article/**Supplementary Material**.

## Ethics Statement

The studies involving human participants were reviewed and approved by the ethics committee of Beijing Tiantan Hospital, Capital Medical University. The patients/participants provided their written informed consent to participate in this study.

## Author Contributions

WM: data analysis and editing the manuscript. ZB: data collection and organization of CGGA database. YZ: data collection and organization of TCGA database. KZ: draw the figures. TJ: conception, supervision, and design of the manuscript. All authors contributed to the article and approved the submitted version.

## Funding

This study was supported by the National Natural Science Foundation of China (81761168038, 81972337); Nature Science Foundation of Beijing (JQ20030).

## Conflict of Interest

The authors declare that the research was conducted in the absence of any commercial or financial relationships that could be construed as a potential conflict of interest.

## Supplementary Material

Supplementary Figure 1 WHO grade, IDH1 status, MGMT promoter methylation status and transcriptional characteristic subtype relative to *SAMD9* expression. **(A)** The correlation of SAMD9 expression level with WHO grade. SAMD9 expression levels in glioma of WHO grade II-IV in CGGA database 2, *****p* < 0.01. the difference between III & IV, *****p* < 0.0001. **(B)** The relationship between SAMD9 transcription level and *IDH1* mutation in CGGA database 2. *****p* < 0.0001. **(C)** The correlation of SAMD9 expression level and MGMT promoter methylation status, the difference in CGGA database 2, *****p* < 0.0001. **(D)** The relationship between SAMD9 transcription level and *IDH1* mutation in each transcriptional characteristic subtype in CGGA database 2.

Supplementary Figure 2 Patients with higher *SAMD9* level exhibit poorer OS. **(A)** Half of the patients with higher SAMD9 expression exhibited shorter OS in Kaplan-Meier analyses based on CGGA database 2 ALL, *p* < 0.0001. **(B)** The half of patients with higher SAMD9 expression exhibited shorter OS in Kaplan-Meier analyses based on CGGA database 2 LGG, *p* = 0.0002. **(C)** Half of patients with higher SAMD9 expression exhibited shorter OS in Kaplan-Meier analyses based on CGGA database 2 GBM, *p* = 0.058.

Supplementary Figure 3 Univariate and Multivariate Cox regression analyses and correlations with classic genetic alterations of SAMD9. **(A)** Univariate and Multivariate regression analyses of SAMD9 expression level and several related clinical variables in CGGA database 2 LGG, Red color indicates protective factor HR > 1, Green color indicates harmful factors HR < 1, Gray color represents HR crossing 1. HR of SAMD9 and Grade > 1, *p* < 0.0001. HR of *IDH* mutation < 1, *p* < 0.01. HR of 1p/19q codeletion < 1, *p* < 0.001. **(B)** Univariate and Multivariate regression analyses of SAMD9 expression level and several related clinical variables in GBM CGGA database 2, HR of *IDH* mutation < 1, *p* < 0.05.

Supplementary Figure 4 Nomogram prediction model for OS and other integrated risk factors. A nomogram was developed by integrating the SAMD9 expression with the clinicopathologic features, grade, IDH status, 1p/19q codeletion status in the CGGA database 2 (left). Calibration plot of nomogram for predicting OS at 1- (blue line), 3- (red line) and 5- (black line) years, ideal line as control (white line) in the CGGA database 2 (right).

Supplementary Figure 5 SAMD9 expression comparation between normal brain and difference grade gliomas. Total of 207 normal brain tissues, 518 low-grade gliomas, and 207 high-grade gliomas were included in the comparisons. *p* <0.05 LGG & Normal, *p* <0.05 GBM & Normal.
