# Supplementary figures and images for: SAMD9 Is Relating With M2 Macrophage and Remarkable Malignancy Characters in Low-Grade Glioma
Source: Front Immunol. 2021 Apr 16;12:659659. doi: 10.3389/fimmu.2021.659659 (PMC8085496; doi:10.3389/fimmu.2021.659659)

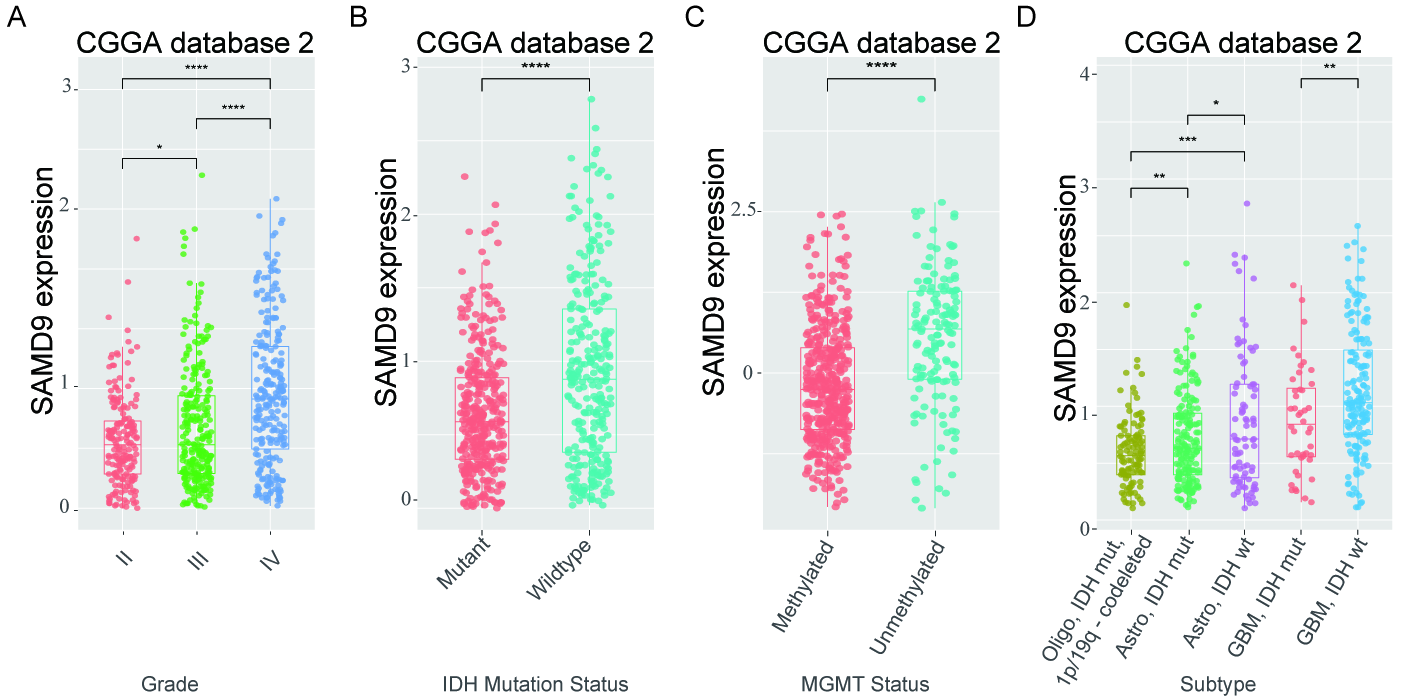

Supplement: Supplementary Figure 1 — WHO grade, IDH1 status, MGMT promoter methylation status and transcriptional characteristic subtype relative to SAMD9 expression. (A) The correlation of SAMD9 expression level with WHO grade. SAMD9 expression levels in glioma of WHO grade II-IV in CGGA database 2, ****p < 0.01. the difference between III & IV, ****p < 0.0001. (B) The relationship between SAMD9 transcription level and IDH1 mutation in CGGA database 2. ****p < 0.0001. (C) The correlation of SAMD9 expression level and MGMT promoter methylation status, the difference in CGGA database 2, ****p < 0.0001. (D) The relationship between SAMD9 transcription level and IDH1 mutation in each transcriptional characteristic subtype in CGGA database 2. [file Image_1.tif]

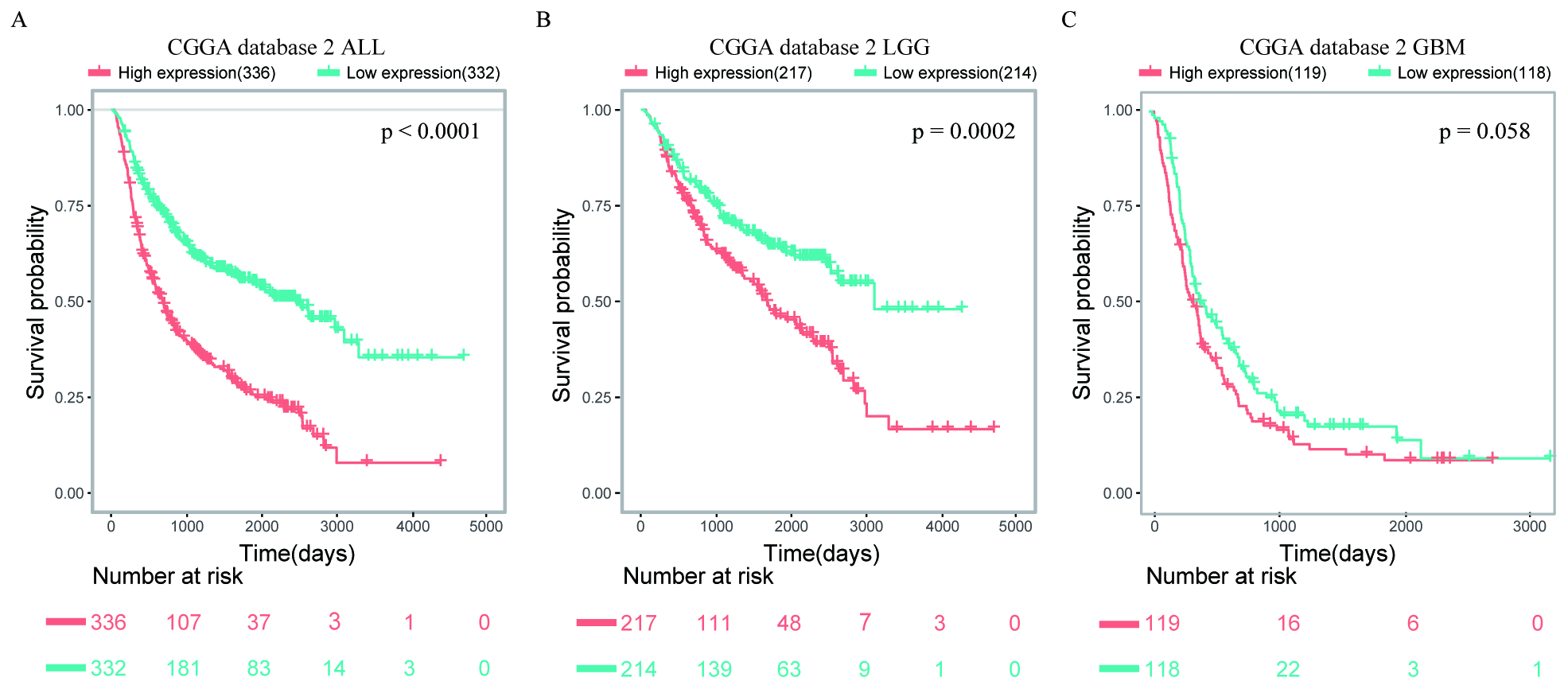

Supplement: Supplementary Figure 2 — Patients with higher SAMD9 level exhibit poorer OS. (A) Half of the patients with higher SAMD9 expression exhibited shorter OS in Kaplan-Meier analyses based on CGGA database 2 ALL, p < 0.0001. (B) The half of patients with higher SAMD9 expression exhibited shorter OS in Kaplan-Meier analyses based on CGGA database 2 LGG, p = 0.0002. (C) Half of patients with higher SAMD9 expression exhibited shorter OS in Kaplan-Meier analyses based on CGGA database 2 GBM, p = 0.058. [file Image_2.tif]

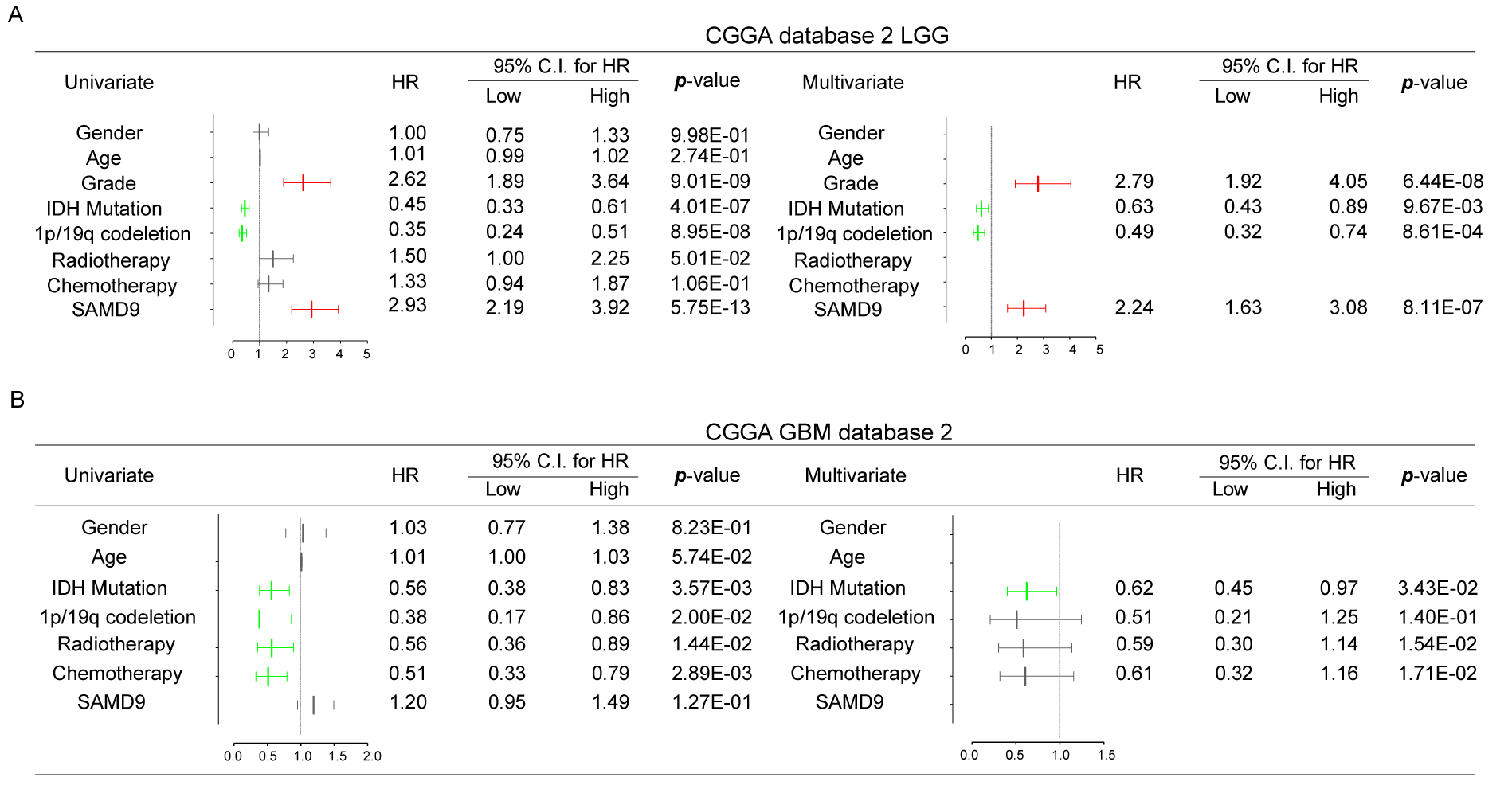

Supplement: Supplementary Figure 3 — Univariate and Multivariate Cox regression analyses and correlations with classic genetic alterations of SAMD9. (A) Univariate and Multivariate regression analyses of SAMD9 expression level and several related clinical variables in CGGA database 2 LGG, Red color indicates protective factor HR > 1, Green color indicates harmful factors HR < 1, Gray color represents HR crossing 1. HR of SAMD9 and Grade > 1, p < 0.0001. HR of IDH mutation < 1, p < 0.01. HR of 1p/19q codeletion < 1, p < 0.001. (B) Univariate and Multivariate regression analyses of SAMD9 expression level and several related clinical variables in GBM CGGA database 2, HR of IDH mutation < 1, p < 0.05. [file Image_3.tif]

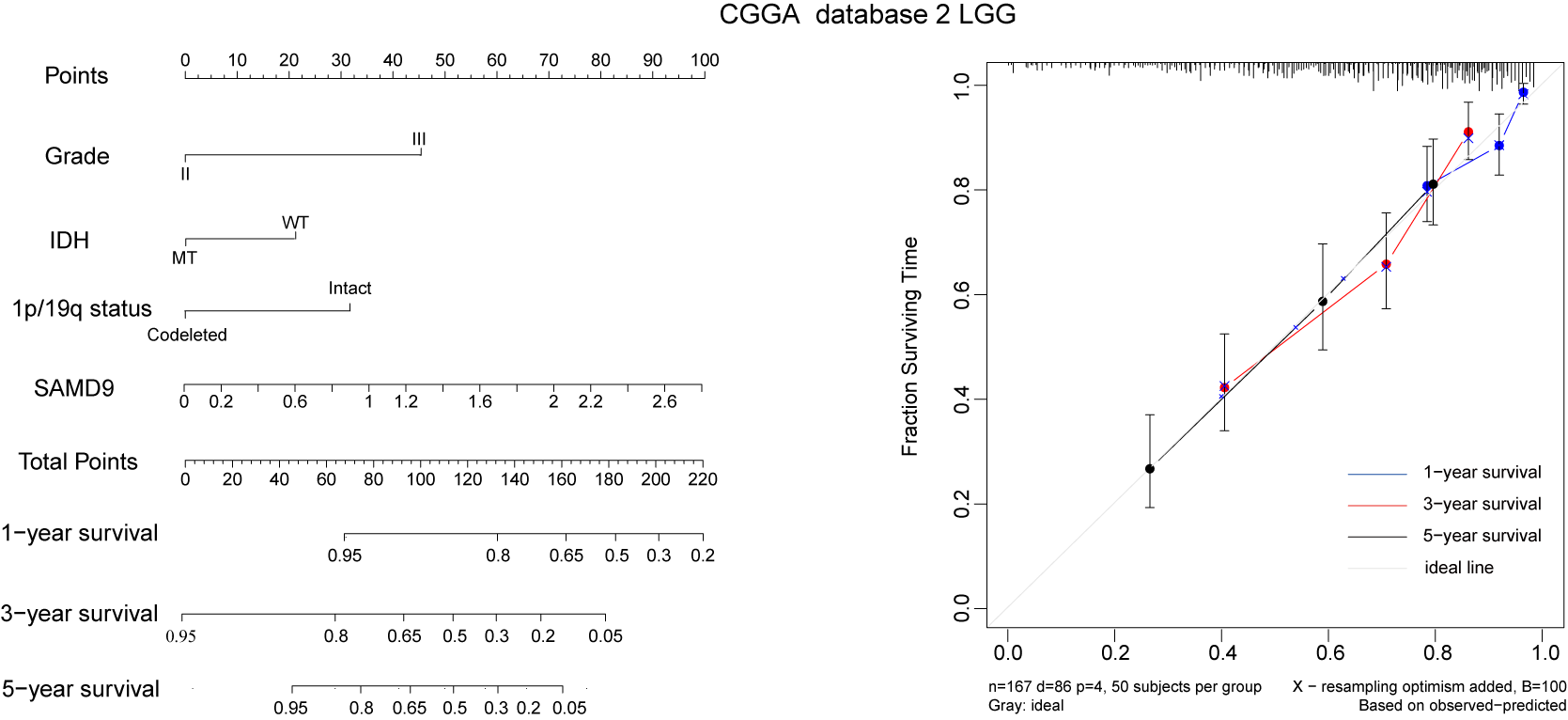

Supplement: Supplementary Figure 4 — Nomogram prediction model for OS and other integrated risk factors. A nomogram was developed by integrating the SAMD9 expression with the clinicopathologic features, grade, IDH status, 1p/19q codeletion status in the CGGA database 2 (left). Calibration plot of nomogram for predicting OS at 1- (blue line), 3- (red line) and 5- (black line) years, ideal line as control (white line) in the CGGA database 2 (right). [file Image_4.tif]

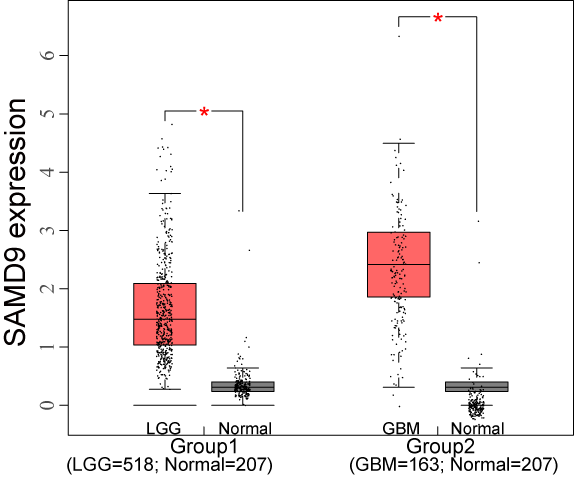

Supplement: Supplementary Figure 5 — SAMD9 expression comparation between normal brain and difference grade gliomas. Total of 207 normal brain tissues, 518 low-grade gliomas, and 207 high-grade gliomas were included in the comparisons. p <0.05 LGG & Normal, p <0.05 GBM & Normal. [file Image_5.tif]
